# Barriers to successful implementation of prevention-of-mother-to-child-transmission (PMTCT) of HIV programmes in Malawi and Nigeria: a critical literature review study

**DOI:** 10.11604/pamj.2014.19.154.4225

**Published:** 2014-10-15

**Authors:** James Christian Okoli, Gail Elizabeth Lansdown

**Affiliations:** 1 Department of Public Health, Faculty of Health and Life Sciences,Oxford Brookes University, OX3 0FL, Oxford, United Kingdom

**Keywords:** Africa, antiretroviral drugs, breastfeeding, HIV/AIDS, Malawi, mother-to-child-transmission, Nigeria, PMTCT, VCT

## Abstract

Mother-to-child-transmission (MTCT) of HIV still remains a significant route of new HIV infection in children in Malawi and Nigeria, despite the introduction of Prevention-of-Mother-to-Child-Transmission (PMTCT) of HIV programmes in both countries. A critical literature review, based on the findings from 12 primary research articles, explores the reasons for the inadequacy and failure of PMTCT. Findings show socioeconomic and sociocultural factors as the biggest barriers to the success of PMTCT programmes. Other factors include: limited male involvement, the organization of PMTCT and health workers’ inefficiency. In conclusion, PMTCT programmes will remain inefficient unless these factors are addressed. There is an urgent need to strengthen PMTCT programmes by stakeholders through a collaborative strategic effort to ensure high PMTCT programme uptake in Malawi and Nigeria, in order to eliminate HIV/AIDS in children.

## Commentary

HIV/AIDS has had a devastating negative effect on women and children alike, accounting for high global maternal and infant/child mortality. In 2009, approximately 370,000 children worldwide were newly infected with HIV and there are 42,000 to 60,000 maternal deaths as a result of HIV/AIDS [1]. Mother to Child Transmission (MTCT) is responsible for the majority of HIV infections in infants and young children below the age of 15 years [2]. Maruet al. (2009) reveal that 40% of these children acquired the infection through breastfeeding making this route the most common for MTCT of HIV [3]. Despite the introduction of PMTCT by WHO, MTCT still remains an important and fast growing route for new HIV infections in sub-Saharan Africa [4].

The annual incidence rate of HIV in Malawi is estimated at 920,000 (830,000 - 1,000,000) with about 1,000,000 adults living with the disease [5]. Prevalence of the disease among pregnant women was estimated at 22.8% in 1999 which declined to 13.5% in 2007 with an estimate of 57,000 women currently living with the disease [6]. In 2009, approximately 58% of pregnant women and 41% of HIV exposed infants received ARV drugs [6]. In Nigeria, only 58% of pregnant women utilize antenatal facilities [7]. Prevalence rates of HIV within this percentage are reported to be 4.6% [7]. Research reveals that about 4.7 million child deliveries take place in Nigeria annually with approximately 75,000 of these infants being born to HIV positive mothers [8]. However, only 22% and 8% of HIV positive pregnant women and HIV exposed infants respectively received ARV drugs in 2009 [7, 9]. As with Malawi, the PMTCT programme in Nigeria still falls short of its targets.

**Figure 1:**
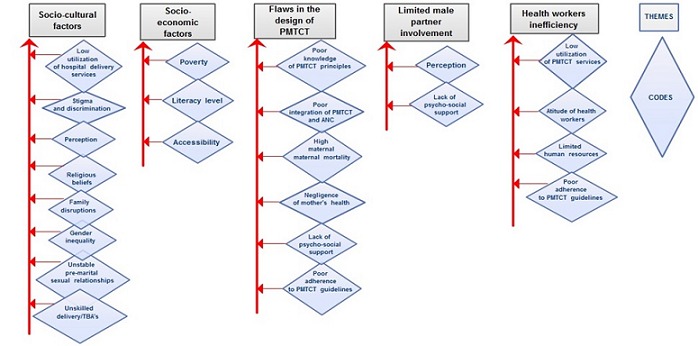
Taxonomic map showing the combination of identical and similar codes to form them

In a bid to contain and eliminate mother to child transmission of HIV in Malawi and Nigeria, the health ministries of both countries assisted by the WHO set up calculated targets to ensure the success of PMTCT by 2013. Both the Malawian and Nigerian health ministries have estimated that approximately 80% of pregnant and lactating women should receive comprehensive PMTCT services by 2013. They also aim to integrate PMTCT programme with maternal and child health services [5, 10]. However, these targets have not been met [8, 11]. A critical literature review of related articles published within the last ten years was conducted. 231 articles were obtained from MEDLINE, CINAHL and WEB OF SCIENCE, with 152, 147 and 72 articles retrieved respectively. A total of 12 articles were found to have fulfilled the inclusion and exclusion criteria and were appraisedusing two critiquing frameworks - Coughlan et al. (2007) [12] for the quantitative studies and Ryan et al. (2007) [13] for the qualitative. Only peer-reviewed literature was selected to ensure high quality and credibility. Thematic analysis was employed using the simplified approach described by Aveyard (2010) [14] to synthesize information embedded in each article. Information from articles was labelled as codes and similar codes were grouped together to develop themes (Figure 1). Initial coding revealed twenty-four codes. Similar codes were collated, reviewed and assigned to themes. Five themes emerged: **THEME 1**: Socio-cultural factors; **THEME 2**: Socio-economic factors; **THEME 3**: Flaws in PMTCT designs; **THEME 4**: Limited male partner involvement; **THEME 5**: Health workers insufficiency.

The results from this study show socioeconomic and sociocultural factors as the biggest barriers to the success of PMTCT programmes. Other factors such as limited male involvement, flaws in the design of PMTCT and health workers inefficiency were also identified (as shown in Table 1). In this study, socio-cultural factors appear to be the most important barrier preventing the successful implementation of PMTCT services in Malawi and Nigeria having been identified in nine of the reviewed articles. This factor is made up of numerous minor challenges such as stigma and discrimination, perception, religious beliefs, family disruption, gender inequality, unstable pre-marital sexual relationships, unskilled birth delivery by Traditional Birth Attendants (TBAs) and low utilization of hospital delivery services. Stigma and discrimination appear to be the most important socio-cultural barrier faced by PMTCT and are reported in all the literature chosen for this study. This was discovered to be the underlying reason why women enrolled in PMTCT programmes fail to disclose their HIV status to their partners and family and in some cases prevent women from enrolling into the programme [15]. Studies reveal cases of physical abuse and violent reactions from partners and families of women enrolled in PMTCT programme which in turn led to their refusal to adhere to PMTCT therapies, disruption of their marriages and/or drop out from the programme [15–20]. Also, cultural norms in these countries afford very limited rights to women to the extent that they often lack the right to make important decisions regarding their own health [21, 22]. In the study carried out in an antenatal clinic in eastern Nigeria, the authors noted that the reproductive health actions of women in that region are often decided by their partners or extended family members, who in most cases prefer to go to TBAs [16, 20, 23].

**Table 1:** Thematic matrix of the appraised articles

ID	Authors	Socio-cultural factors	Socio-economic Factors	Flaws in PMTCT design	Limited male partner involvement	Health workers inefficiency
1.	Balogun and Odeyemi (2010)		**ü**			
2.	Moses *et al*. (2009)	**Ü**	**ü**			
3.	Chinkonde *et al*. (2010)	**ü**		**ü**		**ü**
4.	Galadanci *et al*. (2008)	**ü**		**ü**		
5.	Kasenga *et al*. (2009)	**ü**	**ü**	**ü**		
6.	Levy *et al*. (2010)	**ü**	**ü**	**ü**		**ü**
7.	Njunga *et al*. (2010)	**ü**			**ü**	
8.	O'Gorman *et al*. (2010)	**ü**			**ü**	**ü**
9.	Okonkwo *et al*. (2007)	**ü**	**ü**		**ü**	
10.	Onah *et al*. (2008)			**ü**		
11.	Onah *et al*. (2007)		**ü**	**ü**		
12.	Van Lettow *et al*. (2010)	**ü**		**ü**	**ü**	**ü**
Total		9	6	7	4	4

Secondly, socioeconomic factors are identified as the second biggest barrier limiting the success of PMTCT in both Malawi and Nigeria. Codes under this theme includes: poverty, illiteracy, poor awareness and poor accessibility to PMTCT programmes. Poverty cuts across other codes and themes, where it can be seen as an underlying factor. It is identified as the reason why pregnant women failed to access PMTCT services, as most of them live far away from the hospitals and cannot afford to transport themselves to the hospital for delivery thereby being unable to access not only skilled delivery but also PMTCT therapies and services [17]. Also the choices women make within the PMTCT programmes are affected to a great extent by their socioeconomic status^22^. According to findings from studies, the failure of HIV-positive mothers to adhere to the replacement feeding therapy, choice of traditional birthing methods via TBAs and their preference of vaginal delivery to caesarean was due to lack of awareness and poor socioeconomic status [16, 23, 24]. Flaws in the design of PMTCT are another barrier identified in this study. PMTCT strategies are unarguably the best method to reduce the high prevalence of HIV/AIDS in children [25]. However, the prevalence of this disease in children especially in sub-Saharan Africa still remains a global threat [1]. The inability of PMTCT programmes to cater for maternal needs both in and out of hospital units isreported in both Malawian and Nigerian studies [16, 19, 26, 27]. The authors reveal that the programme is predominantly focused on infant care thereby neglecting the mothers. Studies conducted in Nigeria and in Malawi reveal that pregnant womenwere not given ARV drugs and their CD4 cell count could not be ascertained; therefore resulting in high maternal mortality [18, 27]. Also, PMTCT programmes still lack proper integration with maternal health services such as antenatal and family support units in hospitals. As a result, many HIV-positive women are not reached or are lost to follow-up [19, 21]. In addition, there have been difficulties in adapting to changes in PMTCT policies and guidelines by health workers due to the failure of PMTCT programmes to provide sufficient replacement feeding supplement for infants. This makes it difficult for health workers to propose PMTCT therapies to mothers [19].

Furthermore, limited male partner involvement in PMTCT activities is another barrier common to both countries. PMTCT programmes are considered to be a woman-child affair and do not seem to accommodate any form of male participation hence making male partners oblivious of the gains of the programme [15]. Socio-cultural and socio-economic factors such as literacy and cultural/religious beliefs can also influence low male involvement in PMTCT programmes as noted in a study [17]. The authors report that cultural norms in Malawi make it very difficult for husbands to play significant roles in the reproductive health of their wives, as such decisions are normally left to the elderly women in the family [17]. A Nigerian study reveals that the acceptance of voluntary counselling and testing (VCT) by pregnant women will increase if they are tested simultaneously with their partners [20]. As a result of low male partner involvement in PMTCT, the enrolled women are left to carry the burden of their HIV status and the new lifestyles proposed to them by the programme and are obviously denied the very important psycho-social support by their partners and families [18]. Hence, these women tend to shy away from the programme therefore promoting MTCT of HIV [18].

Finally, the inefficiency of healthcare workers is another barrier encountered in this review. PMTCT programmes have suffered a series of set-backs due to inadequate human resources and under-trained healthcare professionals. Research findings show that PMTCT workers lack proper understanding of PMTCT principles and often fail to give quality counselling to women enrolled in the programme [16]. The authors further reveal that the failure of women to adhere strictly to the six months exclusive breastfeeding option proposed by PMTCT programmes is due to the poor quality counselling they receive [16–18, 20]. This is also as a result of their lack of understanding of the medico-scientific information on how breastfeeding practice can reduce the risk of transmission of the disease to their babies. Therefore most of the women believe that nevirapine will prevent transmission rather than exclusive breastfeeding [16]. Downplaying the effect of the recommended feeding policy means that their babies stand the risk of being re-exposed to the virus. In addition, the lack of consensus on a national guideline for PMTCT programmes has resulted in health workers lacking the courage to strictly advise mothers on the best infant feeding options [19]. As a result, PMTCT workers adhere only to the guidelines with which they are conversant in order to retain the trust of the mothers.

## Conclusion

In conclusion, the study shows that low PMTCT programme efficiency experienced in Malawi and Nigeria is caused by the factors discussed above. Sociocultural and socioeconomic factors prove to be the most important barriers to the successful implementation of PMTCT of HIV services in these two countries. Stigmatisation and poverty, which are sub-factors of sociocultural and socioeconomic factors respectively, were discovered to be vital to the ineffectiveness of PMTCT programmes. Other barriers analysed in the study include: flaws in PMTCT design, limited male partner involvement and inefficiency of healthcare workers.

PMTCT service and its current guidelines developed by the World Health Organization in 2006 still remain the best strategy to eliminate MTCT of HIV but require appropriate application and the strategic implementation of interventions to tackle the barriers [4]. Addressing the issue of inadequate resources both monetary and human still remains vital to the success of the programme. The government of these countries in collaboration with its health sectors, Non-governmental Organizations and various international and local organizations must ensure that more resources are channelled or invested into maternal health services to optimize the operation of PMTCT services and increase resource allocation to the health sector. Eliminating cultural barriers will be achieved by education and re-orientation of communal beliefs and doctrines, which further supports the need to make resources available to ensure communal education/awareness and strict implementation of policies that will aid in eliminating MTCT in Malawi and Nigeria. In addition, improving male partner involvement can be achieved through the initiation of father or husband support groups, in which the male partners of these women are taught exclusively about the benefits of PMTCT principles and the essential support required of them by their partners.

Lastly, it is necessary to ensure there is sufficient availability of multi-disciplinary healthcare providers, who are well trained and well paid, to ensure efficient delivery of PMTCT services. This will support an increased integration of PMTCT programmes and maternal health services to ensure early detection of women in need of the service, thus avoiding the losses to follow up reported in most PMTCT programmes. This will promote the coverage of the service as more women will be reached. Further research is needed to examine the effect of current PMTCT guidelines on other communities. Additionally, research should be carried out to develop innovative educational strategies that are adapted to socio-cultural beliefs of communities to promote acceptance of PMTCT.
